# Changes in the Sodium Content in Branded Foods in the Slovenian Food Supply (2011–2020)

**DOI:** 10.3390/nu15194304

**Published:** 2023-10-09

**Authors:** Sanja Krušič, Hristo Hristov, Maša Hribar, Živa Lavriša, Katja Žmitek, Igor Pravst

**Affiliations:** 1 Nutrition Institute, Koprska Ulica 98, SI-1000 Ljubljana, Slovenia; sanja.krusic@nutris.org (S.K.); hristo.hristov@nutris.org (H.H.); masa.hribar@nutris.org (M.H.); ziva.lavrisa@nutris.org (Ž.L.); katja.zmitek@vist.si (K.Ž.); 2 VIST–Faculty of Applied Sciences, Gerbičeva Cesta 51A, SI-1000 Ljubljana, Slovenia; 3 Biotechnical Faculty, University of Ljubljana, Jamnikarjeva 101, SI-1000 Ljubljana, Slovenia

**Keywords:** food reformulation, sodium, salt, food policy, Europe, Slovenia

## Abstract

High sodium intake is the leading diet-related risk factor for mortality globally. Many countries have introduced policies to support the reformulation of foods and to reduce sodium intake, mainly on a voluntary basis, but there are limited data available about the long-term efficiency of such measures. Slovenia implemented salt reduction policies for the period of 2010–2020; these policies also included the voluntary reformulation of foods with the lowering of sodium content. This study’s aim was to explore the nationally representative branded food datasets collected in the years 2011, 2015, 2017, and 2020 to investigate the changes in the sodium content in prepacked branded foods. The study was conducted with datasets collected from food labels using standard food monitoring studies and included all the major retailers. Differences in market shares were adjusted by sales weighting, which was conducted using the yearly sales data provided by the major retailers. The food categories with a major contribution to the overall sales of sodium in prepacked branded foods were processed meat and derivatives (19.0%), canned vegetables (7.1%), water (6.7%), bread (7.2%), and cheese (6.3%). Considering the available food products, a notable decreasing sodium content trend was observed in biscuits, breakfast cereals, pizza, and spreads. Year-to-year differences were much less expressed after the correction for market share differences, and neutral trends were most frequently highlighted. This indicates that sodium was less frequently reduced in market-leading products. The study results revealed that very limited progress in sodium food reformulation was achieved in the 10-year period, indicating the need for more efficient policy approaches. The study demonstrated the importance of the systematic monitoring of the food supply for the evaluation of food policies.

## 1. Introduction

The high intake of sodium is a worldwide public health concern. Excess sodium intake is one of the leading risk factors for hypertension, while hypertension-related cardiovascular diseases (CVDs) are the leading cause of death from non-communicable diseases [1]. Each year, 17.9 million people die due to CVDs, accounting for 32% of all deaths globally [2]. According to the Global Burden of Disease Study, high sodium intake worldwide is the leading diet-related mortality risk factor, and it is responsible for millions of deaths and disability-adjusted life years (DALY) [3].

The World Health Organization (WHO) recommends that adults should consume less than 5 g of salt/day, which is equivalent to 2 g of sodium per day. However, the typical salt intake is nearly twice (9–12 g salt/day) that of the upper recommended limit [4,5]. Across Europe, the average salt intake ranges between 7 and 13 g per day [6], and approximately 53% of countries in the WHO European Region reported population salt intakes above WHO recommended maximum intakes [7].

Although the primary source of dietary sodium varies from country to country, a significant proportion of sodium intake comes from processed foods—bread and bakery products, cereal and grains, meat products, and dairy products [5]. In many high-income countries, prepared and packaged foods are the source of 75% of dietary sodium intake [8], and this trend is also increasing in low- and middle-income countries [9].

There is a global effort to reduce salt intake. In 2013, the member states of the WHO pledged to reduce sodium intake by 30% by 2025 [10]. Accordingly, the WHO published a technical package, SHAKE (the SHAKE Technical Package for Salt Reduction), for member states in order to assist the sodium reduction strategies in 2016. The WHO encourages governments to establish salt reduction policies and interventions, including surveillance, the promotion of reformulation by industries, the implementation of accurate labelling, the empowerment of individuals through education and communication, and the establishment of environments to promote healthy eating [11].

It is estimated that decreasing the high dietary salt intake from the current level to the recommended level would prevent about 2.5 million deaths from heart attacks and stroke globally [4]. The salt reduction strategies targeting the lowering of the sodium content of commonly consumed foods are effective ways to reduce the sodium intake of the population [12], and salt reduction strategies are considered to be central to the tackling of non-communicable diseases [8]. In principle, salt reduction strategies can target individuals (education, dietary recommendations, and campaigns) and industries (reformulation and salt substitution), or they can make use of economic approaches, such as tax regulations [13,14,15]. Several countries have implemented salt reduction strategies, which include a variety of activities—from individual actions, like consumer education, to structural actions, such as setting sodium content limits and establishing labelling schemes [13]. A review of studies from various countries reporting the changes in sodium content in packaged food products showed that while many countries have implemented salt reduction interventions to reduce the population’s salt intake, very few have published monitoring and evaluation studies [16].

Comprehensive monitoring of the food supply by collecting data from food labels has been proven to be an efficient method for monitoring the content of key nutrients (such as sodium) in branded foods [16,17,18]. Regular cross-sectional food monitoring studies enable tracking market trends on products’ composition over the years [19], providing useful insights into food reformulation activities.

Slovenia is a Central European country in which high dietary salt intake was reported (about 12 g/day) [20]. The National Action Plan to Reduce Salt was accepted in Slovenia in 2010; it aimed to cut the population’s salt intake to the recommended 5 g daily by 2020 [21,22]. The action plan included a wide set of activities, including raising awareness about the risks of excess salt intake and working with the food industry to gradually reduce salt content in processed foods—on a voluntary basis. Reducing salt intake is also a focus of the Slovenian National Program on Nutrition and Health Enhancing Physical Activity 2015–2025 [23], which provided a recommendation for the food industry to gradually lower salt content in critical categories of processed foods by 3.8–5.8% per year. However, Slovenia still has not yet adopted any mandatory measures towards sodium reduction in foods [15]. With respect to these documents, baseline monitoring of the sodium content in processed foods was conducted in 2011 [24] and 2015 [25] as part of the efforts to continuously monitor the food supply; this was conducted within the Slovenian Composition and Labelling Information System (CLAS) program [26]. Further evaluation was supported within the European Food Nutrition Security Cloud (FNS-Cloud) project (https://www.fns-cloud.eu/; accessed on 3 August 2023), funded by the European Commission HORIZON2020 framework program, with a demonstration of the use of branded food datasets in the sodium reformulation case study.

The objective of the study was to investigate the impact of voluntary food reformulation programs to reduce salt content in processed foods in Slovenia. Salt content trends in prepacked branded foods were explored using food supply data collected between the years 2011 and 2020.

## 2. Datasets and Methods

### 2.1. Datasets, Data Collection, and Processing

This case study was conducted using branded food datasets for Slovenia for the period 2017/2020; it was compared with a previously reported comparison for the period of 2011/2015 [24,25]. The data collection approach is described in detail elsewhere [26]. Briefly, the datasets were generated by a standard food monitoring study of the major food retailers with the largest nationwide shop networks, covering most of the food supply. All available prepacked products were systematically photographed and entered into the CLAS database (Composition and Labelling Information System (CLAS)) [26], using European Article Number (EAN) barcodes as product identifiers. The collection of the food photographs in the food stores was conducted using the mobile application CLAS, while further data processing was conducted using the CLAS web platform. Barcodes were used to avoid duplicate entries. The labelling of pictures was used for the extraction of various parameters; the declared salt content was used for this study. The dataset was further amended with national yearly sales data obtained from the major retailers. The sales data were given in a universal form, including the barcode number, which was used for food matching. The sales data of different suppliers were totalled for each barcode number, giving overall information about the quantity of sold items per year across the country. To enable the comparison with the previous data, the foods were categorised according to the recommendations of the Global Food Monitoring Group (GFMG) [27], with minor adaptations when considering the specifics of the European market. The case study was focused on the following food (sub)categories of prepacked foods: bread; biscuits; cakes, muffins, and pastries; breakfast cereals; pasta; pizza; soups; ready meals; preprepared salads and sandwiches; cheese; butter and margarine; canned vegetables; processed meat and derivatives; meat alternatives; crisps and snacks; sauces; mayonnaise/dressings; spreads; and water. Examples of food items in these food (sub)categories are provided in Supplementary Table S1.

The 2017 sample contained 11,595 products with available sales data, of which 9474 had available sodium content data. Altogether, 62.9% (N = 5962) of these corresponded to the above-mentioned food categories. Furthermore, the 2020 sample contained 12,302 products with available sales data, of which 9147 had available sodium content data. Of these, 73.9% (N = 6759) corresponded to the above-mentioned food categories. The 2011 and 2015 datasets were previously described in detail and were used to estimate the salt content in the specific food (sub)categories [24,25]; the results of these studies were used for descriptive comparisons.

### 2.2. Sodium Content in Foods

The labelled salt content was used to calculate the sodium content of each product in the database. In most cases, the sodium content was calculated for a product in the form in which it was sold (in mg per 100 g or mL of food). The exception was soups, where the sodium content was re-calculated for the prepared soup, using the dissolution factor according to the manufacturer’s labelled recommendation for the preparation of the soup.

Sodium content in the investigated food (sub)categories was then presented using descriptive statistics. All calculations were performed with the inclusion of all foods with both sodium and sales data. For each food sub(category), we calculated the median sodium content (in mg of sodium per 100 g or mL), together with P25 and P75 percentiles. To support comparisons with previous studies [24], we also calculated the mean sodium content (MSC).

The sale-weighting approach [25] was used to account for market-share differences; the sale-weighted sodium content (SSC) was calculated for food (sub)categories using the following formula:

$${SSC}_{for\;category}=\frac{\sum_{i}c_{i}\times m_{i}}{\sum_{i}m_{i}}$$

*c_i_* … sodium content in specific food (*i*), included in the food (sub)category;
*m_i_* … yearly amount of sold specific food (*i*);
*i* … 1 → n (for all products in selected food (sub)category).

We also calculated the ratio between the MSC and SSC values (SAR values).

### 2.3. Major Sodium Sources in Branded Foods 

To provide insights into the major sodium sources among prepacked branded foods, we further calculated ‘Share in total sodium sales’ (STSS) for each investigated food (sub)category using the following formula:

$$S{TSS}_{for\;category}=\frac{\sum_{i}c_{i}\times m_{i}}{\sum_{k}\sum_{i}c_{i}\times m_{i}}$$

*k* … 1 → n (for all included food categories).

STSS, therefore, presents the contribution of each food (sub)category to the total sodium content in all the included sold prepacked foods. It should be noted that the calculated STSS only corresponds to the food categories covered in this study (see Section 2.1), which do not include non-prepacked foods (i.e., non-prepacked bread), which are also considered notable dietary sources of sodium.

### 2.4. Foods with Excessive Sodium Content 

Excessive sodium content in foods was determined using the PAHO criteria [28] for each product in the dataset with labelled sodium and energy content larger than 0 kcal. We calculated the ratio between the amount of sodium (mg) and the energy (kcal) in the product. Products with a ratio equal to or higher than 1 were assigned as high in sodium [28]. The assessment was performed for all categories except for the categories of water and soups. In the next stage, we calculated the crude proportion (High Na; %) and sale-weighted proportion (SW High Na; %) of products with excessive sodium content for each (sub)category.

### 2.5. Data Analyses

The data were processed and analysed using Stata Statistical Software, Release 17.0, for Windows (StataCorp LLC, College Station, TX, USA) and IBM SPSS v.26 and Microsoft Excel 2013 (Redmond, WA, USA), while visualisations were also performed using GraphPad Prism version 10.0.0 for Windows (GraphPad Software, Boston, MA, USA). The median, mean, and 95% confidence intervals (95% CI) were calculated for the sodium content. On the other hand, the sale-weighted sodium content (SSC) was calculated as an exact value. Data analyses for 2017 and 2020 were performed for all above-mentioned food categories, while long-term comparisons (2011–2022) were performed only for major contributors in sodium intake, using a threshold STSS (2020) > 2%. Statistical analyses were not conducted for (sub)categories with less than 25 products in a single collection year. To determine if the medians originate from the same population of products in the selected categories in different years, we conducted a non-parametric k-sample test for the equality of the medians with a continuity correction. Differences in the MSC between the years were evaluated with an independent sample one-tailed *t*-test. *p* < 0.05 was considered statistically significant.

## 3. Results

The largest contributors to the overall sales of sodium (STSS) in prepacked branded foods were processed meat and derivatives (21.4 and 16.8% for 2017 and 2020, respectively); canned vegetables (9.7 and 7.1%); water (6.7 and 5.7%); bread (9.0 and 8.6%); and cheese and imitates (6.6%) (Table 1).

Among the observed food categories, the highest median content of sodium for the year 2020 was found in processed meat and derivatives (800 mg/100 g), followed by crisps and snacks (720 mg), cheese imitates (720 mg), and sauces (680 mg) (Table 1). Most of these categories were also high in sodium after correction for market share differences using the yearly sales data. The highest SSC values were observed for Cheese imitatates (915 mg); crisps and snacks (805 mg); processed meat and derivatives (797 mg); and sauces (768). In most categories, the sodium content increased when market differences were corrected using sale-weighing, indicating higher sodium content in market-leading products. The most notable exception was meat alternatives.

A comparison of the sodium levels in the selected categories of prepacked foods in the Slovenian food supply in 2020 with those of the 2017 data is presented in Table 1 and Supplementary Table S2. In general, the year-to-year differences were less expressed after the market share differences were addressed using sales weighting. A higher sales-weighted sodium content was observed in water (160%), the noodle subcategory (141%), and cheese and imitates (116%). It should be noted that statistically significant differences in median sodium content between both years were only observed for three main categories of processed meat and derivates (0.005), soups (*p* = 0.018), crisps and snacks (0.035), and a subcategory of vegetable spreads (<0.001). A higher median sodium content in 2020 was observed in processed meat and derivates, and a lower one was observed in soups, crisps and snacks, and vegetable spreads.

**Figure 1:**
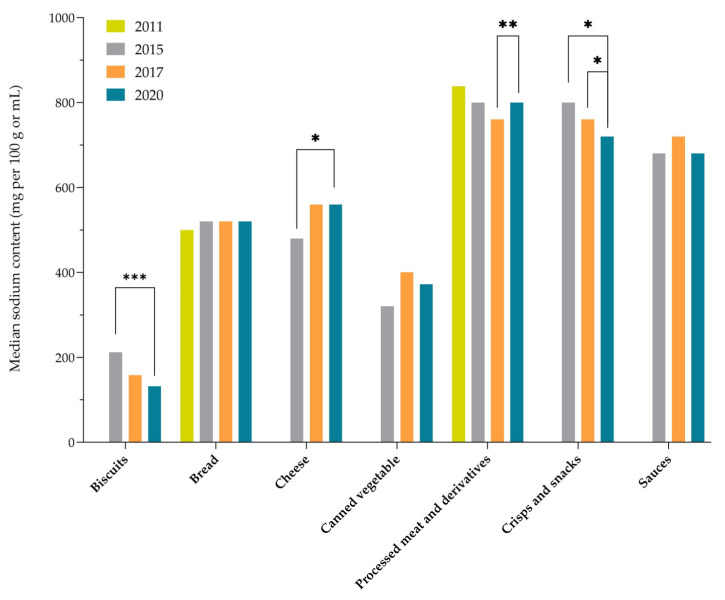
Median sodium content in selected categories of prepacked branded foods in Slovenia (2011–2020). Significant difference between food categories in different years: (***) = *p* < 0.001; (**) = *p* < 0.01; and (*) = *p* < 0.05.

**Figure 2:**
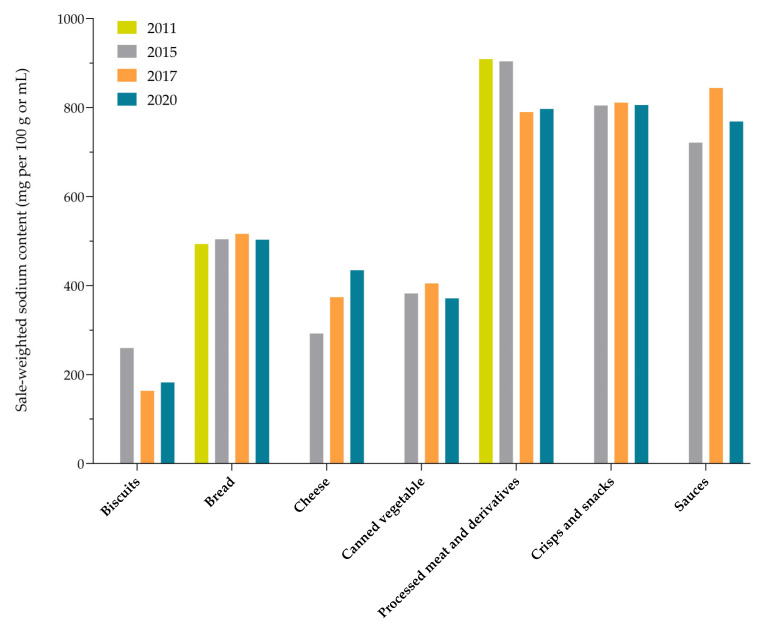
Sales-weighted sodium (SSC) content in selected categories of prepacked branded foods in Slovenia (2011–2020).

To provide longer-term insights into the sodium reformulation trends in Slovenia, the study results were further compared with the results of previous monitoring conducted in the years 2011 [24] and 2015 [25], focusing on the comparison of food (sub)categories that were included in other monitoring studies and for which sufficient data on sodium levels were available. Comparisons were made for the following major contributors in sodium intake (using a threshold STSS (2020) > 2%): biscuits; bread; cheese; canned vegetables; processed meat and derivatives; crisps and snacks; and sauces. The sodium content trends in the branded foods in the years 2011, 2015, 2017, and 2020 are presented in Figure 1 (for median sodium content) and Figure 2 (for sales-weighted sodium content). As presented in Figure 1, statistically significant changes were only observed in biscuits (between 2015 and 2020), cheese (between 2015 and 2020), processed meat and derivatives (2017 vs. 2020), and in crisps and snacks (2015 vs. 2020; and 2017 vs. 2020). A general observation was that the year-to-year differences are mostly less expressed for the sales-weighted data (SSC). In most of the selected food categories, the results of the year-to-year comparison of sales-weighted sodium levels (Figure 2) show quite neutral trends.

We also investigated food category proportions of products with excess sodium using PAHO criteria [28]. Both crude and sale-weighted proportions of foods high in sodium are presented per (sub)category in Table 1. Above 90% of products with excess sodium in 2020 was observed in pizza (100%), plain bread (99%), pasta sauces (98%), cheese imitates (97%), processed meat and derivatives (95%), spreads and processed cheese (94%), canned vegetables (93%), and sauces (92%). Also, after accounting for market-share differences in all these categories (and additionally in ready meals and vegetable spreads), the sale-weighted proportion of high-sodium foods exceeded 90%. The lowest proportion of high-sodium foods (<10%) were found in biscuits (4%), dry pasta (5%), and breakfast cereals (7%); in the latter case, the proportion increased to 16% after sale-weighing.

## 4. Discussion

As mentioned above, among all the selected categories of prepacked foods, processed meats and derivatives were identified as the most important contributors to salt intake (17% in 2020); this was similar to the situation in other countries [29,30]. This food group contributes about one fifth of the sodium intake, as it does in other developed countries [31]. Reducing the level of sodium in processed meats is challenging because this not only affects sensory properties but also impacts shelf life and food safety [31] because the addition of salt increases the osmotic pressure, which reduces water activity and thus produces a bacteriostatic effect [32]. This also affected the trends in consumer behaviours, particularly with regard to the clean-label movement and the demand for naturalness in food products [33,34], which involve limiting the use of food additives. Research has also shown that NaCl can be replaced by KCl by up to 50% without changing the taste and texture of some meat products [35,36]. The WHO target values for this category vary from 230 mg/100 g for raw meat products and preparations (such as unprepared meat products and burgers and fresh sausages) to 950 mg for non-heated, preserved whole muscle meat products (air-dried, cured entire meat pieces, e.g., Parma and Serrano ham, and brined meat products, e.g., bacon) [12]. Considering the observed sodium content for this category, the improvement options are obviously not fully exploited. In comparison with other countries, higher sodium contents were reported in China [37], and lower values were reported in the USA [29], Spain [30], Argentina [38], Brazil [39], and South Africa [40]. It should also be noted that the high consumption of processed meat has also been identified as a risk factor for the development of colorectal cancer [41], meaning that consumers should also be educated with regard to how to reduce the overall consumption of such products, independently of the sodium content.

Cheese is another major contributor to salt intake (2020 STSS: 6.6%). As with processed foods, the reduction in sodium content in this food category is challenging due to the multifaceted role of salt in cheese production [42]; it is used as an emulsifier, and it reduces water activity, improves the flavour of the cheese, and protects it from the development of spoilage bacteria and pathogens [43]. Furthermore, various types of cheese contain different amounts of sodium; this also depends on the ripening, which is reflected in the water content. The sodium content benchmarks set by the WHO [12] encompass the following values: 190 mg per 100 g for fresh unripened cheese, 520 mg for soft/medium ripened cheese, 625 mg for semi-hard ripened cheese, and 510 mg for mould-ripened cheese. In light of these benchmarks, it is noteworthy that sodium content in cheese in Slovenia is considerably elevated, with a median value of 560 mg and a sales-weighted sodium content (SSC) of 597 mg. This sodium content in Slovenian cheese surpasses those found in Argentina [38], South Africa [40], the Czech Republic [44], and Brazil [39], while remaining lower than those observed in Spain [30] and China [37]. It is additionally disconcerting to observe that the sodium content in cheese imitates, when weighted by sales, is even more elevated, reaching 915 mg. While this subcategory currently makes a limited contribution to the overall sodium content in sold prepacked foods, we need to consider the European Commission’s Green Deal sustainability goals and the Farm to Fork Strategy [45], which highlight the move towards more plant-based diets. Plant-based imitations of both meats and cheeses are expected to gain popularity and might become a notable source of sodium in the future.

The high prevalence of daily bread consumption in Slovenia, with the accompanying moderately high sodium content in this food category, has made bread a major source of dietary sodium intake [46]. This was also confirmed in our study (STSS in 2020: 8.6%). It should be noted that this value applies only to prepacked bread, while a high proportion of bread in Slovenia is sold as non-prepacked, meaning that the overall contribution of bread to the sodium intake is even larger. Nevertheless, despite bread being a specific category targeted in the National Action Plan to Reduce Salt [21,22], significant improvements have not been evident since 2011, when the inaugural food monitoring study took place in Slovenia. For instance, in 2020, the sales-weighted sodium content in plain bread stood at 504 mg/100 g, only slightly higher than the 492 mg recorded in 2011. Comparable sodium levels were also observed in non-prepacked foods [46], contrasting with the WHO benchmark for bread, which is set at a mere 330 mg [12]. It is worth noting that notably lower sodium levels were reported in bread in the United Kingdom [17]. Furthermore, the sodium content of bread in Spain has nearly halved within an eight-year span [30,47], although it remains higher than the WHO benchmark, a pattern also observed in Argentina [38], Costa Rica [48], the USA [49], Brazil [39], the Czech Republic [44], and South Africa [40]. In Italy, sodium levels were reported to be nearly twice that of the WHO benchmark [50], whereas China has successfully reduced sodium levels below the benchmark [37]. Considering the high daily intake of bread in Slovenia (177 and 118 g in adult males and females, respectively [51]), it would be worth exploiting further activities to support the reduction in sodium levels. In addition to the pledge of the Slovenian Chamber of Commerce to reduce sodium content in bread voluntarily by 5% in the period of 2019–2020, a more efficient option would be to provide a regulatory upper limit to the sodium levels in plain bread, as was also proposed in the Netherlands [52]. Considering that in Slovenia, a lot of food businesses are operating in this sector, many of which are not members of the Slovenian Chamber of Commerce, such an approach would have much higher potential. In addition, one way to reduce salt in the food chain is based on the fact that human receptors can adapt to low salt concentrations over time; this can be used to compensate for the lower salt content in processed foods because small incremental reductions in salt in products are not sensorily detectable [53]. Recent studies showed that a 15–25% reduction, and sometimes a reduction as significant as 33%, in salt in white bread did not affect consumers’ hedonic preference; this was not the case with multigrain bread [54,55,56]. However, to support policymakers in the monitoring of bread reformulation, future studies should also be focused on non-prepacked bread.

As in the 2015 study [25], bottled water was again highlighted as an important contributor to total sodium sales in prepacked foods (STSS: 5.7%). This category is also a most striking example of the importance of the consideration of sales data in the assessment of the food supply. For example, while the median sodium content in 2020 was 0.76 mg/100 mL, the sale-weighted content was much higher (58 mg/100 mL). It should be noted that while most bottled water products in the Slovenian food supply contain less than 1 mg of sodium per 100 mL, a few products with a very high market share (>60%) contain ≥50 mg/L. It is also interesting that the majority of the category was represented by mineral water (87.4% in 2017 and 76.8% in 2020).

Our study highlighted very limited effects of the current national voluntary reformulation strategy on the sodium content in prepacked foods in the Slovenian food supply. Various factors contribute to the effectiveness of food reformulation policies to improve food composition and enhance dietary patterns and overall public health, including robust incentives and a well-structured implementation plan with monitoring and evaluation mechanisms [57,58]. Rosewarne et al. published a global review of national strategies for sodium reduction in branded foods, showing that out of 62 countries with identified sodium reformulation strategies, 43 countries have delineated sodium targets and only 15 are enforcing mandatory regulations at least to some level [59]. It seems that the mandatory approach is much more efficient in comparison with voluntary programmes. For example, South Africa was one of the early adopters of mandatory regulations aimed at reducing salt content in processed foods in 2016, and recent monitoring studies suggested notable effects on both sodium content in foods [60] and sodium intake in the population [61]. On the other hand, monitoring of the voluntary programs produced very diverse results. Rosewarne et al. investigated changes in sodium levels in packaged foods in Australia between 2014 and 2019 when a media advocacy intervention was implemented to stimulate sodium reformulation, but no meaningful reduction in sodium levels was observed in targeted packaged foods [62]. On the other hand, the United Kingdom implemented a comprehensive voluntary salt reduction program in 2003, encompassing a series of progressively lower, product-specific reformulation targets for the food industry, coupled with consumer advice to reduce salt intake, but the effects of activities were also limited. The slight reduction in the salt content of foods and the total volume of salt sold between 2015 and 2020 did not achieve statistical significance, resulting in a recommendation that large food companies should be required to report their salt sales to enhance transparency regarding individual businesses’ progress toward salt reduction targets [63]. Actually, robust and transparent monitoring seems to be a key point for successful sodium reformulation. Our results highlighted the importance of the consideration of sales data to account for huge market-share differences between products in the food supply. To achieve meaningful changes in the sodium content at the population level, these changes need to be reflected particularly in market-leading products. The observed challenges in this area in Slovenia could be partially explained with a hypothesis that particularly newly launched foods might have lower sodium content in comparison with existing market-leading products, which are rarely reformulated. While such a hypothesis should be investigated with future studies, policymakers could improve the sodium reformulation strategy by setting specific sodium content target limits for key food categories, preferably with a mandatory approach and robust monitoring.

The major strengths of this study are that we were able to analyse data collected in different cross-sectional studies in Slovenia in the period of 2011–2020 and that we had access to nationwide yearly sales data. Exploiting the sales data provided very useful insights into the food supply because this enabled the correction of the very different market shares of the different products. This is particularly relevant in the food supply, where some niche products have an almost neglectable market share, while a very small number of bestselling products can dominate the market. Therefore, the median sodium content in foods available in the markets can obscure the real-life situation, as observed in some food categories in our study.

However, the used approach is also related to a limitation. The sales data were unfortunately not available for all the products, but we had access to data for the majority of the products. This limitation is also highly relevant for all such studies in other regions because some retailers are not willing to share sales data, which are usually considered confidential. Actually, most of the food monitoring studies did not explore sales data at all. The limitation of our comparison is that some previous monitoring studies were conducted with a somewhat different monitoring approach and only on selected food categories. This is particularly relevant for the food monitoring study in 2011, which was conducted using direct data collection in food stores without photographing the food labels, and also in a different regulatory situation, when the sodium content was still voluntary and a notable proportion of food labels were missing this information. Another study limitation is that the sodium content data for this study were taken from food labels and not from laboratory analyses. However, considering that the labelling of the nutrition declaration is strictly regulated [64] and can be subject to the official control of the food authorities, this was the most reasonable approach. Chemical analysis of all the marketed foods is simply not feasible, while a randomised analysis approach would result in other limitations. As food labelling data have proven to be a reliable data source in the scientific studies of other nutrients [65], we have no reason to speculate that the labelling data on sodium content would be less reliable.

## 5. Conclusions

While the voluntary gradual reduction in the sodium levels in processed foods in Slovenia was recommended back in 2010, our study revealed limited progress among prepacked foods, at least in food categories with a major contribution to dietary sodium intake. This is an indication that the implementation of more efficient public health strategies would be beneficial. In addition to consumer education and collaboration with the food industry for reformulation activities, a measurable approach would be to set specific sodium content target limits for specific food categories. To ensure the widest implementation of such target limits, these should be regulated—at least for the key contributors to sodium intake, such as bread. Possible targets of regulatory salt limits are public procurement, food-labelling instruments, and a mandatory salt content limit when substantiated by public health requirements.

## Figures and Tables

**Table 1:** Sodium content in prepacked foods for selected food (sub)categories in 2017 and 2020.

Food Factory	2020									2017									Median Diff. 2020/2017 *p*-Value	SSC Ratio 2020/2017 (%)
	N	% LSC	Sodium Content (mg per 100 g/mL)				High Na (%)	SW High Na (%)	STSS (%)	N	% LSC	Sodium Content (mg per 100 g/mL)				High Na (%)	SW High Na (%)	STSS (%)		
			P25	Med.	P75	SSC						P25	Med.	P75	SSC					
Bread	413	93	440	520	720	504	90	97	8.6	361	98	440	520	700	517	89	96	9	0.499	97
- Plain bread	169	91	440	480	520	486	99	100	6.3	134	97	440	480	520	489	95	99	5.3	0.855	100
Biscuits	770	98	64	132	240	182	4	3	2.3	707	95	80	152	260	163	5	3	3	0.091	112
Cakes, muffins, and pastries	376	98	92	188	320	191	17	12	0.9	344	92	80	180	280	214	13	18	1.4	0.477	90
Breakfast cereals	418	99	16	96	212	193	7	16	1.0	320	95	16	100	254	218	13	22	1.4	0.654	89
Pasta	744	97	4	10.2	110	123	18	20	1.8	633	94	4	12	88	129	21	22	2.2	0.916	96
- Noodles	62	98	12	40	400	370	23	40	0.5	87	92	16	40	720	263	29	28	0.7	0.866	141
- Dry pasta	569	96	2.8	4	40	30	5	2	0.3	445	95	2.8	5.2	32	31	5	3	0.4	0.393	96
Pizza	25	100	440	520	560	494	100	100	0.1	26	100	480	514	572	523	100	100	0.2	0.921	94
Soup (prepared)	197	100	312	360	400	369	NA	NA	0.5	206	99	338	374	409	380	NA	NA	0.9	0.018 *	97
Ready meals	212	83	288	400	520	442	88	98	1.4	187	89	320	400	520	441	98	100	2	0.886	100
Preprepared salads and sandwiches	85	94	356	528	600	511	85	82	0.5	39	95	360	520	600	530	97	100	0.4	0.400	96
Cheese and imitates	523	98	300	560	760	434	79	62	6.6	475	96	320	560	800	374	83	56	5.6	0.176	116
- Cheese	302	98	400	600	760	597	81	82	4.7	266	97	400	600	760	568	88	79	3.4	0.236	105
- Spreads and processed cheese	135	98	320	440	890	570	94	97	1.5	155	96	350	480	90	554	93	98	1.8	0.905	103
- Cheese imitates ^a^	33	100	680	720	920	915	97	100	0.1										NA	NA
Butter and margarine	91	95	4	40	200	65	14	1	0.4	92	91	4.2	42	200	77	8	1	0.6	0.885	84
- Canned vegetables	497	100	200	372	608	371	93	96	7.1	515	93	244	400	780	405	95	96	9.7	0.099	92
Processed meat and derivatives	732	92	640	800	1320	797	95	94	16.8	703	91	580	748	1000	780	94	96	21.4	0.005 **	102
Meat alternatives	122	98	176	544	760	324	76	53	0.1	90	93	60	352	700	353	69	51	0.1	0.089	92
Crisps and snacks	432	100	520	720	960	805	78	87	4.8	340	98	600	768	988	811	86	83	5.7	0.035 *	99
Sauces	686	98	440	680	1200	768	92	94	4.9	581	91	440	720	1280	844	94	99	6.4	0.486	91
- Pasta sauces	131	100	400	532	800	541	98	100	0.8	149	89	400	520	920	740	94	100	1	0.807	73
Mayonnaise/dressings	76	99	400	560	600	467	40	7	0.9	70	100	440	560	640	466	40	12	1.3	0.874	100
Spreads	265	97	40	200	480	142	43	19	0.4	174	90	48	332	560	143	47	15	0.5	0.469	99
- Vegetable spreads	104	96	400	472	520	467	86	99	0.2	60	93	480	560	600	540	95	99	0.3	0.001 **	86
Waters	95	88	0.28	0.76	5.1	58	NA	NA	5.7	99	88	0.24	0.76	4.8	36	NA	NA	6.7	0.946	160

Notes: % LSC: percentage of products with labelled sodium content; Med.: median; SSC: sale-weighted sodium content in prepacked foods; STSS: share in total sodium sales; High Na: proportion of foods (%) with excess sodium content, according to the PAHO criteria [28], that was applied to SSC; SW high Na: sale-weighted sodium proportion of foods (%) with excess sodium content, according to the PAHO criteria; NA: not applicable. ^a^ Statistical comparison was not conducted for cheese imitates due to small sample size in 2017. (*) *p* < 0.05; (**) *p* < 0.01.

## Data Availability

The datasets generated for this study are available upon request to the corresponding author.

## Supplementary Materials

The following supporting information can be downloaded at https://www.mdpi.com/article/10.3390/nu15194304/s1. Table S1: Examples of foods included in the investigated (sub)categories; Table S2: Sodium content in prepacked foods for selected food (sub)categories in 2017 and 2020.
