# Supplementary material for: Changes in the Sodium Content in Branded Foods in the Slovenian Food Supply (2011–2020)
Source: Nutrients. 2023 Oct 9;15(19):4304. doi: 10.3390/nu15194304 (PMC10574098; doi:10.3390/nu15194304)
Supplement: Supplementary file 1 [file nutrients-15-04304-s001.zip › nutrients-2569554-supplementary.pdf]

nutrients

Supplementary Materials

Changes in the Sodium Content in Branded Foods in the Slovenian Food Supply (2011–2020)

Sanja Krušič [1], Hristo Hristov [1], Maša Hribar [1], Živa Lavriša [1], Katja Žmitek [1,2] and Igor Pravst [1,2,3,*]

Table S1. Examples of foods included in the investigated (sub)categories.

Food categories	Examples of included foods
Bread	Plain bread, crispy bread, tortillas, dough
- Plain bread	Wheat bread, baguette, bread crumbs, toast, mix grain breads, small bread products, corn mixed bread, rye mixed bread, etc.
Biscuits	Sweetened pastries and biscuits, brioche, butter biscuits, wafers, cookies, ...
Cakes, muffins, and pastry	croissants, croissants with filling, cakes, muffins, etc.
Breakfast cereals	Corn flakes, sweet flakes, cokolino, sport muesli, wholemeal, rolled cereals/oat flakes, puffed rice, other cereals
Pasta	pasta, noodles, whole grain pasta, etc.
- Noodles	noodles
- Dry pasta	All kinds of dry pasta
Pizza	Pizza
Soup (prepared)	Soup concentrates, canned soups, etc.
Ready meals	Prepared dishes, lasagna paella, nuggets, pasta dishes, etc.
Preprepared salads and sandwiches	Salads, sandwiches, sushi, fish salads, potato salads, french salads; stuffed baguettes, garlic or butter bread, tortillas (wrap)
Cheese and imitates	All kinds of soft and hard cheese, cheese spreads, cheese imitates, etc.
- Cheese	All kinds of soft and hard cheese, curd, cottage cheese
- Spreads and processed cheese	Cheese spreads and processed cheese
- Cheese imitates	Cheese-like vegetable products
Butter and margarine	Butter and margarine
Canned vegetables	Canned beets, peas, beans, corn, etc.
Processed meat and derivatives	Salamis, pate, frankfurters, sausages, etc.
Meat alternatives	Meat-like vegetable products
Crisps and snacks	Savoury snacks, chips, etc.
Sauces	Pasta sauces, sweet and savoury sauces
- Pasta sauces	Bolognese sauce, pesto sauce, cream sauce, etc.
Mayonnaise/dressings	Mayonnaisse, salad dressings, etc.
Spreads	Chocolate spreads, peanut butter, vegetable spreads, hummus, etc.
- Vegetable spreads	Hummus, soy spreads, vegetable spreads, etc.
Water	Bottled water (with or without gas)

Table S2. Sodium content in prepacked foods for selected food (sub)categories in 2017 and 2020.

Food (sub)category	2020			2017				
	N	Sodium content (mg per 100g/mL) Mean [MSC] (95% CI)	SAR MSC/SSC Ratio (%)	N	Sodium content (mg per 100g/mL) Mean [MSC] (95% CI)	SAR MSC/SSC Ratio (%)	SCA Mean diff.(SE) 2020/2017	SSC Ratio 2020/2017 (%)
Bread	413	566 (347;784)	89	361	559 (349;769)	93	−7 (15.4)	97
- Plain bread	169	483 (380;587)	101	134	489 (315;663)	100	6 (16.1)	100
Biscuits	770	169 (−9;347)	108	707	186 (35;337)	88	17 (8.6)*	112
Cakes, muffins and pastry	376	221 (55;387)	87	344	207 (51;363)	103	−14 (12.0)	90
Breakfast cereals	418	157 (−66;380)	123	320	191 (−59;441)	114	34 (17.5)*	89
Pasta	744	133 (−154;420)	93	633	144 (−168;456)	89	11 (16.2)	96
- Noodles	62	257(−76;590)	144	87	317 (−235;869)	83	60 (78.7)	141
- Dry pasta	569	76 (−194;346)	40	445	55 (−152;262)	56	−21 (15.5)	96
Pizza	25	520 (417;623)	95	26	564 (386;743)	93	44 (40.9)	94
Soup (prepared)	197	352 (272;432)	105	206	375 (293; 457)	101	23 (8.0)*	97
Ready meals	212	536 (−133;1205)	82	187	428 (291; 565)	103	−108 (49.8)[x]	100
Pre-prepared salads and sandwiches	85	507 (269;745)	101	39	464 (322; 606)	114	−43 (41.2)	96
Cheese and imitates	523	603 (29;1177)	72	475	576 (217; 936)	65	−27 (30.7)	116
- Cheese	302	680 (3;1356)	88	266	637 (300; 975)	89	−43 (45.8)	105
- Spreads and processed cheese	135	584 (269;899)	98	155	605 (270; 939)	91	21 (38.3)	103
- Cheese imitates[a]	33	789 (518;1059)	116	3	1027 (980; 1073)	100	238 (158.4)	NA
Butter and margarine	91	178 (−94;451)	36	92	144 (−74; 363)	54	−34 (36.5)	84
- Canned vegetable	497	623 (−401;1647)	60	515	740 (−488; 1967)	55	117 (71.2)*	92
Processed meat and derivatives	732	1073	74	703	895 (391; 1398)	87	−178 (96.6)[x]	102
Meat alternatives	122	500 (145;854)	65	90	438 (68; 808)	81	−62 (50.2)	92
Crisps and snacks	432	754 (384;1124)	107	340	815 (424; 1206)	99	61 (27.5)*	99
Sauces	686	1280 (−397;2957)	60	581	1381 (−453;3214)	61	101(98.7)	91
- Pasta sauces	131	721 (132;1309)	75	149	851 (−296; 1999)	87	130 (111.3)	73
Mayonnaise/dressings	76	545 (346;744)	86	70	557 (352; 762)	84	12 (33.5)	100
Spreads	265	292 (−100;684)	49	174	345 (9; 682)	42	53 (36.2)	99
- Vegetable spreads	104	452 (233;671)	103	60	593 (289; 896)	91	141 (41.0)*	86
Waters	95	16 (−21;53)	357	99	14 (−25; 52)	267	−2 (5.4)	160

Notes: MSC: Mean sodium content of available prepacked foods (95% confidence interval); SCS: Sale-weighted sodium content in prepacked foods (data in Table1); SAR: Ratio between MSC and SCS; [a] category for which statistical analysis was not conducted, due to small sample size; * Significant decrease in MSC ($P < 0.05$). [x] Significant increase in MSC ($P < 0.05$).
